# The profile of severe asthmatics: Results from a specialized asthma clinic

**DOI:** 10.5578/tt.20239919

**Published:** 2023-06-13

**Authors:** Z.Ç. SÖZENER, B.Ö. ÖZTÜRK, Ö. AYDIN, D. MUNGAN, S. BAVBEK

**Affiliations:** 1 Division of Immunology and Allergic Diseases, Department of Chest Diseases, Ankara University Faculty of Medicine, Ankara, Türkiye

**Keywords:** Severe asthma, asthma phenotypes, allergy, eosinophilia, asthma onset

## Abstract

**ABSTRACT:**

The profile of severe asthmatics: Results from a specialized asthma clinic

**Introduction:**

In patients with severe asthma, individualized treatment, and appropriate phenotyping are required to achieve control. In our study, our aim was to examine the characteristics of a specific patient group in a specialized tertiary asthma outpatient clinic, which is the primary setting for evaluating severe asthma patients, with the intention of obtaining national data.

**Materials and Methods:**

In this cross-sectional observational study, sociodemographic, clinical presentations, laboratory results, and spirometry measurements of patients with severe asthma who were followed up in our specialized asthma outpatient clinic for at least one year were recorded. Patients were defined as eosinophilic if they had a blood eosinophil count of 300/µL or higher at least twice during the oral corticosteroid free-period or 150/µL or higher under oral corticosteroids as allergic if they had sensitization to at least one inhalant allergen consistent with their history.

**Results:**

Overall, 201 severe asthma patients (74.1% female) with a median disease duration of 15 (min-max= 1-49) years and a median follow-up duration of 7 (min-max= 1-40) years were analyzed. Most of the patients (56.7%) had adult-onset asthma [median age of onset was 32 (min-max= 10-62) years]. Overweight and obese patients were in the majority (31.8%, and 41.8%, respectively) and the median body mass index was 29 (min-max= 17.5-49.5). More than half of the patients (55.2%) had controlled asthma and the median Asthma Control Test score at the last visit was 23. Biologic therapies were applied to 73.1% (n= 147) of the patients [60.5% (n= 89) omalizumab, 39.5% (n= 58) mepolizumab]. Half of the group was allergic (49.3%) and three-quarters of them were eosinophilic (72.1%). Allergic patients had earlier asthma onset and had more controlled disease than nonallergic ones. Eosinophilic patients were younger and less obese than noneosinophilic patients. Obese and late-onset asthmatics had more uncontrolled disease than normal weight subjects and early onset patients.

**Conclusion:**

The high rate of disease control in the patients with severe asthma in the current study demonstrated the importance of targeted individualized therapy with accurate phenotyping in specialized asthma outpatient clinics.

## Abstract (tr)

**ÖZ:**

Özelleşmiş bir astım polikliniğinin ağır astım verileri

**Giriş:**

Ağır astımlı hastalarda kontrolü sağlamak için bireyselleştirilmiş tedavi, bunun için de uygun fenotiplendirme gereklidir. Çalışmamızda, ülkemizde ağır astımlı hastaların en çok görüldüğü özelleşmiş üçüncü basamak bir astım polikliniğinde bu hasta grubuna ait özellikleri incelemeyi ve bu yolla ulusal veri elde etmeyi amaçladık.

**Materyal ve Metod:**

Kesitsel ve gözlemsel olan çalışmamızda astım polikliniğimizde en az bir yıldır takipli olan ağır astımlı hastalarımızın sosyodemografik ve klinik özellikleri, laboratuvar sonuçları ve spirometrik ölçümleri dosyalarından kaydedildi. Kortikosteroid almadıkları dönemde en az iki ölçümde periferik kan eozinofil sayıları >300 hücre/µL olan hastalar veya kortikosteroid tedavisi altında >150 hücre/µL ve üzeri hastalar eozinofilik olarak kabul edildi. Klinik öyküleri ile uyumlu en az bir inhalan allerjen duyarlılığı olan hastalar ise allerjik olarak tanımlandı.

**Bulgular:**

Ortanca hastalık süresi 15 (min-maks= 1-49) yıl ve polikliniğimizdeki ortanca takip süresi yedi (min-maks= 1-40) yıl olan toplam 201 ağır astımlı hastanın (%74,1’i kadın) verileri analiz edildi. Hastaların yarısından fazlasının (%56,7) erişkin başlangıçlı astımı vardı ve ortanca astım başlangıç yaşları 32 (min-maks= 10-62) yıldı. Aşırı kilolu ve obez hastalar çoğunluktaydı (sırasıyla %31,8; %41,8) ve ortalama vücut kitle indeksleri 29 (min-maks= 17,5-49,5) idi. Önemli oranda hastanın (%55,2) astımı kontrol altındaydı ve çalışma grubunun son vizitteki ortanca astım kontrol testi skoru 23 idi. Biyolojik tedaviler ağır astımlı hastaların %73,1’ine (n= 147) uygulanmıştı [%60,5 (n= 89) omalizumab, %39,5 (n= 58) mepolizumab]. Hastaların yaklaşık yarısı allerjikti (%49,3) ve dörtte üçü eozinofilikti (%72,1). Allerjik hastalar daha erken başlangıçlı astıma sahipti ve non-allerjik olanlara göre astım kontrol düzeyleri daha iyiydi. Eozinofilik hastalar eozinofilik olmayanlara göre daha gençti ve daha az obezdi. Obez hastaların ve geç başlangıçlı astımı olanların astım kontrol düzeyleri, normal kilolu ve erken başlangıçlı astımlılarınkine göre daha kötüydü.

**Sonuç:**

Ağır astımlı hastalardaki yüksek hastalık kontrol oranı; özelleşmiş astım polikliniklerinde doğru fenotiplendirme ile hedefe yönelik bireyselleştirilmiş tedavinin önemini göstermektedir.

## Introduction

Severe asthma, which affects 3-10% of all patients with asthma, is associated with poor asthma control, frequent asthma exacerbations, and increased use of healthcare services, and is therefore responsible for the high disease and economic burden on societies ( 1 , 2 ). Accordingly, it is important to diagnose severe asthma correctly, determine the subtype of this heterogeneous disease, and provide personalized treatment after excluding complicating factors ( 1 ). Especially in the last decade, clinical phenotyping and inflammatory endotyping have gained currency in identifying suitable patients for current treatment options, particularly in identifying candidates for specific biologic therapies ( 3 , 4 ).

Age, sex, body mass index (BMI), onset of asthma, atopy status, exacerbation frequency, and triggers such as smoking, non-steroidal anti-inflammatory drugs (NSAIDs), and exercise are key criteria in the phenotyping process. Accordingly, five phenotypes were identified in the severe asthma research program (SARP) cohort: the early-onset atopic, mild-tomoderate group, the obese late-onset non-atopic group with frequent exacerbations, the highly variable allergic severe asthma group, and the severe airflow obstruction group using oral corticosteroid (OCS) therapy ( 5 ). On the other hand, three severe asthma phenotypes were identified in the U-BIOPRED cohort based on clinical variables. The first group consisted of smokers or ex-smokers with late-onset asthma and chronic airflow obstruction, the second group consisted of non-smokers with chronic airflow obstruction using OCS therapy, and the third group consisted of obese women with frequent exacerbations and normal pulmonary function ( 6 ). Although there are complex inflammatory pathways, mainly two specific endotypes (T2 high and low/non-T2) have been defined to identify therapeutic targets. Eosinophil predominance is observed in T2-high asthma, which includes both allergic eosinophilic and non-allergic eosinophilic asthma ( 7 ). Currently available targeted monoclonal antibodies are directed against T2 inflammation ( 8 , 9 ). Furthermore, it is also recommended to consider specific T2 asthma phenotypes including eosinophilic granulomatosis with polyangiitis (EGPA), allergic broncho-pulmonary aspergillosis (ABPA), and NSAID-exacerbated respiratory disease (NERD) in the follow-up and the treatment plan of severe asthmatics ( 1 , 10 ). Non-T2 asthma includes neutrophilic, mixed, or paucigranulocytic inflammation patterns. The mechanisms underlying non-T2 asthma are not fully understood, these patients are less responsive to steroids and, unfortunately, targeted therapies seem difficult for them currently ( 7 , 11 ).

In light of all these data, we can say that detailing the phenotype and endotype of patients with severe asthma and following them in experienced centers are important for the management of the disease process. As a reference specialist asthma follow-up clinic, ( 12 , 13 , 14 , 15 , 16 ) we aimed to define the sociodemographic, clinical, and inflammatory profile of our patients with severe asthma, and reveal the characteristics and course of their treatment and follow-up.

## MATERIALS and METHODS

### Study design

The present study was conducted as a cross-sectional analysis at a reference specialized asthma center in a tertiary-care hospital. All patients’ medical records were reviewed following the acquisition of written consent from the patients and approval from the institutional review board. The study was performed in accordance with the Declaration of Helsinki and with the approval of the local ethics committee (Approval no: i7-422-20).

### Study group and data recording

Patients included in the study needed to meet specific criteria for enrollment, including being diagnosed with severe asthma based on the Global Initiative for Asthma (GINA) 2020 criteria ( 17 ), being 18 years of age or older, and having a minimum of one year of regular follow-up at our clinic. Follow-up visits were performed every 3-6 months, and sometimes more frequently based on the needs of patients, with a standard examination that included assessing treatment adherence and inhaler technique, evaluation of comorbid diseases, and review of environmental and occupational triggers. Patients who had to take step 4/5 asthma treatment to control the disease or remained uncontrolled despite the provision of the correct inhaler technique and correction of deteriorating factors such as comorbidities and exposure to triggers, were considered to have severe asthma ( 17 ).

Sociodemographic features (age, sex, smoking habit, educational status), phenotypic and clinical presentations (atopy, eosinophilia, asthma onset, obesity), presence of comorbidities, allergic bronchopulmonary aspergillosis (ABPA), eosinophilic granulomatosis with polyangiitis EGPA, or NERD were recorded. Spirometry measurements, peak absolute eosinophil count, number of planned or unplanned visits, asthma exacerbations, hospitalizations, emergency admissions, use of OCS, and history of biologic treatment were also noted.

### Classification of the subjects

Patients with a blood eosinophil count of 300/μL or higher on at least two occasions during the period without oral corticosteroid (OCS) use, or 150/μL or higher while receiving OCS during the follow-up period, were classified as having eosinophilic asthma. Those whose absolute eosinophil count on at least three measurements did not reach 300/μL during the OCS-free period or 150/μL under OCS were classified as non-eosinophilic ( 1 ). Patients were defined as allergic if they were sensitive to at least one inhalant allergen consistent with their history and clinical features in skin prick tests and/or specific IgE measurements ( 1 ). The patients were divided into four phenotypes according to allergy and eosinophilia status: allergic-eosinophilic (AE), non-allergiceosinophilic (NAE), allergic-non-eosinophilic (ANE), non-allergic-non-eosinophilic (NANE).

According to the American College of Rheumatology (ACR) criteria, patients were diagnosed as having EGPA if they had four of six criteria: asthma, eosinophilia greater than 10% in differential white blood cell count, mononeuropathy or polyneuropathy due to a systemic vasculitis, paranasal sinus abnormalities, migratory or transient pulmonary opacities, and evidence of histological eosinophilic vasculitis or perivascular eosinophilic infiltration or eosinophil-rich granulomatous inflammation ( 18 ).

The diagnosis of ABPA was made by combining the following clinical, radiologic, and immunologic signs: bronchial asthma, A. fumigatus skin test positivity or elevated specific IgE levels, elevated total IgE levels (greater than 417 kU/mL), radiologic pulmonary opacities, and elevated total eosinophil counts ( 19 ).

Patients diagnosed as having asthma before the age of 18 years were defined as early-onset, between the age of 18 and 40 years as adult-onset, and after the age of 40 years as late-onset ( 20 , 21 ). According to BMI values (kg/m2), patients were categorized into three groups (normal weight= 18.5-24.9, overweight= 25-29.9, and obese= ≥30) ( 22 ).

We used three main control factors in the standard approach to the assessment of asthma control at routine visits: asthma symptom control, history of asthma exacerbations in the previous year, and variability of forced expiratory flow in one second (FEV_1_) in spirometry measurements ( 1 ). Patients with an Asthma Control Test (ACT) scores of ≥20, FEV_1_ variability of less than 12% between visits in the past year, and no history of asthma exacerbation requiring systemic steroid use in the previous year were evaluated as well-controlled. Patients were considered partially controlled if one of these three conditional factors could not be reached. Those who did not meet any of these three criteria were defined as uncontrolled.

Comorbidities present in the patients were classified as allergic or systemic. Allergic rhinitis, food allergy, urticaria, latex allergy, and atopic dermatitis were categorized as allergic comorbidities. Hypertension (HT), diabetes mellitus, coronary artery disease, thyroid diseases, anxiety-depression, migraine, renal failure, gastroesophageal reflux disease (GERD), chronic sinusitis, nasal polyposis, and bronchiectasis were systemic comorbidities. All comorbidities were diagnosed, treated, and followed by the relevant specialist.

#### Measurements

Allergic sensitization was measured by performing skin prick tests with common aeroallergen extracts (Dermatophagoides pteronyssinus; Dermatophagoides farinae; mixtures of grass pollens, weed pollens, tree pollens, and cereal pollens; molds; and cat and dog epithelia) (ALK, Abello, Spain) and/or testing specific IgE using a CAP fluoroenzyme immunoassay (Phadia, Uppsala, Sweden). Prick tests were considered positive if at least 3 mm or more edema (accompanied by erythema) occurred in the area where the allergen was applied in early readings 15 minutes after the test. Histamine (10 mg/mL) and negative control (saline) were used for validation. The CAP fluoroenzyme immunoassay system (Phadia, Uppsala, Sweden) was used for specific IgE testing. The cut-off value of specific IgE concentrations for sensitization was defined as 0.35 kU/L.

Spirometric measurements [FEV_1_, forced vital capacity (FVC), peak expiratory flow (PEF), and maximum mid-expiratory flow (MMF)] were performed using a spirometry device (ZAN 100, Germany) and evaluated according to the American Thoracic Society/European Respiratory Society (ATS/ERS) guidelines ( 23 ).

##### Statistical Analysis

The Statistical Package for the Social Sciences (SPSS for Windows, version 21.0, SPSS Inc., Chicago, IL, USA) was used to perform the statistical analyses. Descriptive statistics for nominal data are presented as counts and percentages, and for quantitative data either as mean ± standard deviations or medians and minimum-maximum depending on assumptions of normality, which were evaluated using visual (histogram and probability graphs) and analytical methods (Kolmogorov-Smirnov/Shapiro-Wilk tests). The significance of the difference between the means in the normally distributed groups was calculated using analysis of variance (ANOVA), and the significance of the difference between the median values in the non-normal distribution groups was calculated using the Mann-Whitney U or KruskalWallis test. Values below 0.05 were considered significant for all p-values.

## RESULTS

A total of 201 patients with severe asthma (74.1% females, 25.9% males) with a median disease duration of 15 (min-max= 1-49) years and median follow-up duration of seven (min-max= 1-40) years were included in the study. The median age of the patients was 52 (min-max= 19-78) years. Nearly half of the study group was allergic (49.3%) and nearly three-quarters were eosinophilic (72.1%). Most of the group (56.7%) had adulthood-onset asthma and the median age of onset was 32 (min-max= 10-62) years. Overweight and obese patients were in the majority (31.8% and 41.8%, respectively) and the median BMI of the study group was 29 (min-max= 17.5- 49.5). Only two patients were smokers ( Table 1 ).

According to the asthma control evaluation in the last one year; 55.3% of patients had controlled asthma and the median ACT at the last visit was 23. In general, the patients attended follow-up visits regularly and the median number of scheduled visits was 6 (min-max= 0-24). The median number of unscheduled visits, exacerbations, hospitalizations, and emergency admissions was negligible. Biologic therapies were scheduled for 73.1% (n= 147) of the patients. Omalizumab was scheduled for 60.5% of the 147 patients, and mepolizumab was planned for 39.5%. Some 35.8% of the subjects did not need systemic corticosteroids in the last year, 31.8% needed systemic corticosteroids 1-3 times per year, and 32.3% regularly used oral corticosteroids at a median dose of 3 mg (min-max= 1-16 mg) ( Tables 1 and 2 ).

**Table 1:** Demographic characteristics of the patients

Gender % (n)	Women	74.1 (149)
	Men	25.9 (52)
Age median (min-max)		52 years (19-78)
Allergy, % (n)	Allergic	49.3 (99)
	Non-allergic	50.7 (102)
Blood eosinophilia, % (n)	Eosinophilic (≥300 cell/µL)	72.1 (145)
	Non-eosinophilic (<300 cells=""/µL)	27.9 (56)
	Obese (BMI≥ 30)	41.8 (84)
Obesity, % (n)	Overweight (BMI= 25-29.9)	31.8 (64)
	Normal (BMI< 25)	26.4 (53)
BMI, median (min-max)		29 (17.5-49.9)
Age of asthma onset, median (min-max)		32 years (10-62)
Asthma onset, % (n)	Early onset	9.5 (19)
	Adult onset	56.7 (114)
	Late onset	33.8 (68)
Disease duration, median (min-max)		15 years (1-49)
Follow-up duration, median (min-max)		7 years (1-40)
History of smoke, % (n)	Non-smoker	78.1 (157)
	Current Smoker	20.9 (42)
	Ex-smoker	1 (2)
Biologic treatment (Omalizumab/Mepolizumab), % (n)	None	26.9 (54)
	Omalizumab	60.5 (89)
	Mepolizumab	39.5 (58)

BMI: Body mass index

**Figure 1:**
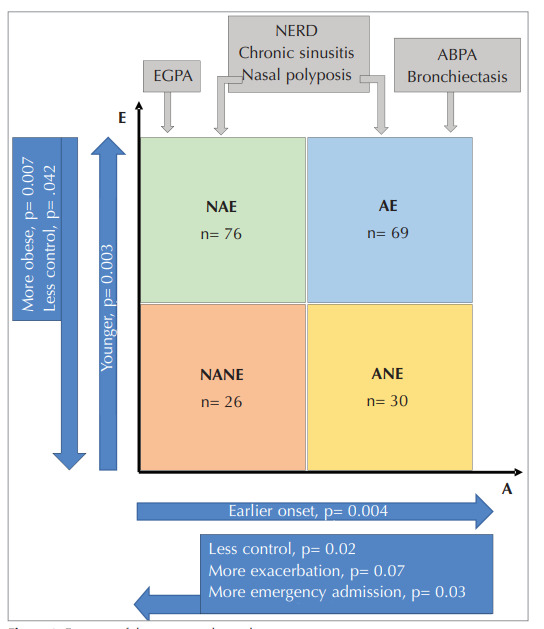
Features of the severe asthma phenotypes. AE: Allergic-eosinophilic, NAE: Nonallergic-eosinophilic, ANE: Allergic-noneosinophilic, NANE: Nonallergic-noneosinophilic, N: Number of patients, EGPA: Eosinophilic granulomatosis with poliangiitis, ABPA: Allergic bronchopulmonary aspergillosis, NERD: Non‐ steroidal anti‐inflammatory drugs‐exacerbated respiratory disease

Among allergic patients, 26.4% were monosensitized. House dust mite was the most common allergen. When patients were grouped according to eosinophilic and/or allergic status, there were 69 (34.3%) patients with AE, 76 (37.8%) with NAE, 30 (14.9%) with ANE, and 26 (12.9%) patients with NANE. Patients with NAE were younger, and patients with ANE had earlier onset of asthma than those with NANE (p= 0.003 and p= 0.004, respectively). Although there was no significant difference between these four phenotype groups in terms of obesity, noneosinophilic patients were found to be more obese than eosinophilic patients (BMI= 30.53, BMI= 27.47, p= 0.007, respectively) ( Figure 1 ).

When the control status was evaluated according to disease phenotypes, patients with non-allergic severe asthma were less controlled than allergic patients (52.9% and 36.4%, respectively, p= 0.02). Albeit not significant, non-eosinophilic patients with severe asthma tended to be less controlled than eosinophilic patients (50% and 42.8%, respectively, p= 0.42) ( Figure 1 ). The rates of uncontrolled patients in the AE, ANE, NAE, and NANE phenotype groups were 33.3%, 43.3%, 51.3%, and 57.7%, respectively (p= 0.08). Although the asthma control status of the patients was the same, patients with NANE and NAE tended to have more exacerbations and emergency admissions than patients with AE and ANE in the last year (exacerbation frequency: 0.69, 0.74, 0.47, and 0.43, p= 0.07, respectively; the number of emergency admissions: 0.27, 0.11, 0.07, and 0.07, p= 0.03, respectively) ( Figure 1 ). Patients under biologic treatments had better-controlled disease than patients not receiving biologics (62.58% vs. 37.41%, p= 0.001)

Furthermore, obese and overweight patients with severe asthma were more frequently uncontrolled than patients with normal weight (54.8%, 48.8%, and 23.5%, respectively, p= 0.004). Age of asthma onset was younger in patients with controlled severe

**Table 2:** Control parameters of the study group in the last one-year period

Asthma control status %	Well controlled	49.8 (100)
(n)	Partly controlled	5.5 (11)
	Uncontrolled	44.8 (90)
ACT, median (min-max)		23 (7-25)
Pulmonary Function tests	Min FEV_1_-Max FEV_1_ lt	1.68 (0.35-4.17)-2.16 (0.59-5.51)
Median(min-max)	Min FEV_1_-Max FEV_1_ %	71 (13-132)-90 (26-132)
	Min FEV_1_/FVC-Max FEV_1_/FVC	71 (35-90)-76 (42-96)
Number of scheduled visits		6 (0-24)
Median (min-max)		
Number of unscheduled visits		0.00 (0-2)
Median (min-max)		
Number of asthma exacerbations		0.00 (0-5)
Median (min-max)		
Number of hospitalizations		0.00 (0-1)
Median (min-max)		
Number of emergency visits		0.00 (0-3)
Median (min-max)		
Systemic steroid treatment requirement % (n)	None	35.8 (72)
	1-3 per/year	31.8 (64)
	Regularly	32.4 (65)

ACT: Asthma control test, FEV_1_: Forced expiratory volume in one second, FVC: Forced vital capacity.

asthma than in uncontrolled subjects [30 (min-max= 10-62), 36 (min-max= 14-60), respectively, p= 0.009]. In terms of biologics, patients under omalizumab treatment were mostly AE, whereas patients on mepolizumab were NAE in general (p< 0.001). In our severe asthma group, treatment steps were generally constant (87.6%), we seldom made step-up or step-down treatment (10.4% and 2%, respectively), and this situation was not found to be related to being allergic and or eosinophilic, obesity, age of asthma onset, using biologics, or having low FEV_1_.

In terms of comorbidities, nasal polyposis, allergic rhinitis, HT, NSAID hypersensitivity, GERD, and chronic sinusitis were most frequently seen (34.3%, 31.8%, 25.5%, 25.4%, 24.4%, and 23.4%, respectively). Diabetes mellitus, osteoporosis, and glaucoma were seen in nearly 13% of patients with severe asthma. We had 23 patients with severe asthma with bronchiectasis and 65.2% were allergiceosinophilic (p= 0.008). Patients with nasal polyposis or chronic sinusitis mostly had AE or NAE asthma (49.3% and 40.6%, respectively, p< 0.001; 44.7% and 48.9%, respectively, p= 0.003). For NERD (n= 43), 48.8% of the patients were non-allergiceosinophilic, and 41.9% were allergic-eosinophilic (p= 0.01). Patients with EGPA (n= 27) were mostly in the NAE phenotype (63.7%), and 86.7% of the patients with ABPA had AE asthma (p= 0.003 and p< 0.001, respectively) (Figure 1).

## DISCUSSION

Our study population consisted predominantly of non-smokers, and overweight or obese women, with adult late-onset and well or partial-controlled severe asthma. They were mostly eosinophilic and nearly half were allergic. About a quarter of the patients used OCS regularly and a high proportion received biologic therapies. Among the study population, patients with NAE were in the majority, followed by AE. Although the four phenotypic groups were similar regarding control, non-allergic patients were found to be less controlled than allergic patients, and patients with NAE and NANE had more asthma exacerbations and emergency admissions. Obese and overweight patients were more uncontrolled than patients with normal weight. Nasal polyposis

and allergic rhinitis were the most frequent comorbidities in the population, followed by HT, NSAID hypersensitivity, GERD, and chronic sinusitis.

The female predominance seen in our severe asthma population was consistent with previous studies. ( 24 , 25 ) The sex imbalance in asthma and severe asthma is thought to be related to the effects of sex hormones on airway epithelium and inflammatory cells ( 26 , 27 ).

Similar to different studies in the literature, overweight or obesity was quite prevalent in our group ( 28 , 29 ). In a cluster analysis of a large number of patients with difficult-to-treat or severe asthma, 57.3% of adolescents and adults were obese ( 30 ). In the data of the British Thoracic Society Difficult Asthma Registry, the proportion of obesity was about 48% in patients with severe asthma ( 31 ). In another severe asthma cohort, 79.29% of patients were obese ( 32 ). Moreover, obesity appeared to be associated with severe asthma and uncontrolled disease. In a study of 492 patients, obesity was found to be an independent risk factor for worse asthma control in women with severe asthma ( 33 ). Another study with a large sample size also demonstrated that obesity was associated with uncontrolled asthma ( 34 ). In addition, prior studies presented a connection between obesity and an increased risk of asthma exacerbations and asthmarelated hospitalizations ( 35 , 36 ). Concordant with the literature, the frequency of being uncontrolled was higher among our obese and overweight severe asthmatics.

Several studies demonstrated that the eosinophilic phenotype was common in severe asthmatics. Although our results indicated the same outcome, the rate of eosinophilic patients was 72.1%, which was higher than in previous studies. In the SARP III cohort, the proportion of severe asthmatic adults with a blood eosinophil count ≥300 cells/μL was 38.5% ( 20 ). In a multicenter study from Brazil, the prevalence of the eosinophilic phenotype among patients with severe asthma was 40% using a blood eosinophil count limit of 300 cells/μL, and 73% using a limit of 150 cells/μL ( 37 ). In a study comparing two asthma cohorts, ProAR and U-BIOPRED, rates of severe eosinophilic asthmatics were approximately 38% ( 38 ). In another study with a cut-off value of 200 cells/μL for blood eosinophil counts, the rate of being eosinophilic in severe asthma was 53% ( 25 ). As an asthma specialist reference follow-up clinic, as can be seen from the high number of patients receiving biologic therapy, many patients with asthma from across the country present to our clinic for biologic therapy. This may explain the high number of eosinophilic patients. In addition, the higher rate of patients with eosinophilia in our study may also be due to the high number of patients with EGPA and ABPA. Moreover, the number of patients with chronic rhinosinusitis and nasal polyposis was quite high, which supports the association of chronic rhinosinusitis and nasal polyposis with eosinophilic inflammation in patients with severe asthma ( 39 )

Different from the common knowledge of the strong association of eosinophilia and poor asthma control, ( 40 ) there was no significant effect of being eosinophilic on asthma control in our study. However, in a recent study similar to ours, there was no difference in asthma control among patients with severe asthma with blood eosinophil counts above 300 cells/μL and those below 300 cells/μL ( 37 ), and in Belgian data in severe asthmatics, high blood and/ or sputum eosinophil counts were not associated with asthma control ( 25 ).

In our study population, non-allergic patients had worse control than allergic patients. In addition, severe non-allergic eosinophilic/non-eosinophilic asthmatics had more asthma exacerbations and emergency admissions. This result may be explained by the fact that the vast majority of the patients received biologic treatment and more than half of those who did not receive biologic treatment were non-allergic. According to the data from an Italian registry of severe/uncontrolled asthma, non-allergic patients needed higher doses of ICS to achieve control and more frequently had uncontrolled disease and asthma-related hospitalizations ( 41 ). Additionally, the authors noted that receiving anti-IgE therapy was associated with better asthma control and fewer asthma exacerbations, independent of allergic status ( 41 ).

Our findings demonstrated a significant difference in the age of asthma onset between controlled and uncontrolled groups; controlled patients had earlier asthma onset compared with uncontrolled patients. Consistent with our results, early-onset asthmatics tended to be better controlled, had less healthcare requirement, and used OCS less frequently, in both the SARP and the UBIOPRED training cohorts ( 5 , 6 ). The fact that these patients also tend to be atopic ( 5 , 6 ) and are candidates for a good response to antiIgE therapy ( 1 ) makes the situation more understandable.

Our study has some strengths and limitations. Regular and standardized follow-up, including control of treatment adherence and inhaler technique, is essential in diagnosing severe asthma ( 1 ), and this criterion needed to be met in our study in the specialized asthma outpatient clinic. In addition, to the best of our knowledge, this is the first study from our country in which the data of patients with severe asthma were evaluated comprehensively.

Although our study had a retrospective pattern, the data were derived from our specialized asthma outpatient clinic, as did our previous studies, which was a strength of our study ( 12 , 13 , 15 ). Apart from the blood eosinophil count, other type 2 inflammation markers including fractional exhaled nitric oxide and sputum eosinophil count were not examined because they are not available in our center. However, the fact that blood eosinophil count is a frequently used and reliable marker in determining type 2 inflammation may mitigate this limitation.

## CONCLUSION

In conclusion, determining the characteristics of severe asthmatics for whom conventional treatments are not sufficient is necessary to draw up disease management plans. The present study demonstrated that accurate phenotyping and correct targeted therapy including biologic treatments make it possible to achieve control in individuals with severe asthma.

## Ethical Committee Approval

The study protocol was approved by Ankara University Human Research Ethics Committee (Decision no: İ7-422-20, Date: 13.07.2020).

## Conflict of INTEREST

The authors declare that they have no conflict of interest.

## AUTHORSHIP CONTRIBUTIONS

Concept/Design: SB, DM, ÖA, ZÇS

Analysis/Interpretation: ZÇS, BÖÖ

Data acqusition: All of authors

Writing: All of authors

Clinical Revision: All of authors

Final Approval: All of authors
